# CORM‐A1 delivers carbon monoxide to the kidney and alleviates post‐ischemic renal dysfunction in rat and swine models

**DOI:** 10.14814/phy2.70666

**Published:** 2025-11-14

**Authors:** Roberta Foresti, Sandra Shurey, Qiyue Mao, Hiroaki Kitagishi, Colin J. Green, Roberto Motterlini

**Affiliations:** ^1^ INSERM, IMRB University Paris‐Est Créteil Créteil France; ^2^ Vascular Biology Unit, Department of Surgical Research Northwick Park Institute for Medical Research Harrow UK; ^3^ Department of Molecular Chemistry and Biochemistry, Faculty of Science and Engineering Doshisha University Kyoto Japan

**Keywords:** carbon monoxide (CO), CO delivery, CORM‐A1, proof‐of‐principle, renal ischemia

## Abstract

Carbon monoxide (CO), a gas endogenously produced in mammalian tissues, exerts vasodilatory, anti‐ischemic, and anti‐inflammatory effects. These properties have prompted the development of CO‐releasing molecules (CO‐RMs) for therapeutic purposes. Among this class of compounds is CORM‐A1, a boron‐based carboxylic acid, which generates controlled amounts of CO under physiological conditions. In this proof‐of‐principle study we explored the potential of CORM‐A1 to protect kidneys from warm ischemia and reperfusion (WI/R) injury in rat and swine models. We found that intravenous administration of CORM‐A1 significantly increased blood carboxyhemoglobin (COHb) levels while facilitating CO accumulation in renal tissue, thus confirming its ability to deliver CO to peripheral organs. In rats subjected to 45‐ and 60‐min WI/R, administration of CORM‐A1 improved renal function at reperfusion, as shown by decreased serum creatinine and urea levels. Histopathological analysis revealed substantial protection against tubular damage, cell infiltration, and inflammation, especially after 60‐min ischemia. Protection was dose‐dependent, with higher doses offering enhanced effects. In a swine kidney auto‐transplantation model, CORM‐A1 significantly improved graft function, reduced fibrosis and necrosis, and extended graft survival. These findings position CORM‐A1 as a promising CO prodrug, with translational relevance for clinical applications in kidney transplantation and other ischemia‐related conditions.

## Graphical abstract

CORM‐A1 delivers carbon monoxide (CO) to the kidney and improves renal function following warm ischemia‐reperfusion (I/R) in rats and pigs.

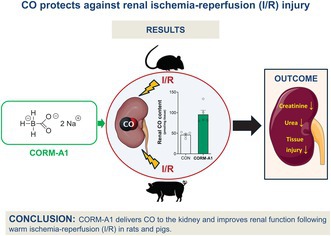

## 1 INTRODUCTION

Carbon monoxide (CO) is a ubiquitous signal transducer continuously produced in mammalian organisms during the degradation of heme by the enzyme heme oxygenase (Lundh et al., 1975; Maines, 1997). Research on the biology of CO has expanded significantly over the last three decades, driven by its ability to exert beneficial effects in a variety of pathological conditions. This gaseous molecule possesses the intrinsic ability to act on multiple cellular targets and coordinate a homeostatic biological response (Motterlini & Foresti, 2017; Szabo, 2010). Initially identified as a vasodilator and neurotransmitter (Furchgott & Jothianandan, 1991; Verma et al., 1993), CO has been documented to exert significant anti‐inflammatory and anti‐ischemic effects in pre‐clinical models of disease, prompting more comprehensive investigations on the use of CO gas as a potential therapeutic in a variety of disorders (Motterlini & Otterbein, 2010; Otterbein et al., 2000; Sato et al., 2001; Yet et al., 2001).

In this context, compounds that have the ability to deliver controlled amounts of CO in vitro and in vivo, known as CO‐releasing molecules (CO‐RMs), have been identified and their chemical reactivity in biological systems duly characterized (Johnson et al., 2003; Motterlini et al., 2002, 2005; Motterlini & Foresti, 2017). CO‐RMs have been extensively documented to recapitulate the pharmacological actions of CO confirming their vasodilatory properties (Fayad‐Kobeissi et al., 2016; Foresti et al., 2004), protective effects against ischemic and inflammatory conditions (Lancel et al., 2009; Wang et al., 2010) and, more recently, have shown efficacy in alleviating obesity and hemolytic disorders in mice (Benrahla et al., 2024; Nguyen et al., 2024). Two major classes of CO‐RMs have been investigated to date: (1) organometallic carbonyls, which consist of CO groups coordinated to transition metals; this class includes compounds containing both transition metals such as manganese (CORM‐401) (Crook et al., 2011) and non‐physiological metals such as ruthenium (CORM‐3) (Clark et al., 2003); and (2) non‐metallic organic prodrugs that can generate CO under specific conditions within cells, tissues and the whole organism (Abeyrathna et al., 2017). Among these is CORM‐A1, a water‐soluble carboxylic acid that contains boron, which has been the first organic CO prodrug to be fully characterized for its spontaneous ability to release CO at physiological conditions in vitro, ex vivo, and in vivo (Motterlini et al., 2005; Ryan et al., 2006; Sandouka et al., 2006).

Previous studies from our laboratory demonstrated that CORM‐A1 could serve as an adjuvant for the preservation of kidney prior to transplantation, indicating that CO could effectively protect this organ from the injurious effects caused by ischemia–reperfusion (Sandouka et al., 2006). In this context, renal damage arises from the cumulative effects of vascular dysfunction, tubular, and glomerular necrosis, and tissue inflammation that may occur before organ harvest, during cold storage, or upon reperfusion after transplantation. Our findings showed that isolated rabbit kidneys flushed and preserved for 24 h with cold Celsior solutions supplemented with CORM‐A1 displayed significantly higher perfusion flow rates, improved glomerular filtration, and enhanced sodium and glucose reabsorption upon reperfusion compared to control untreated kidneys (Sandouka et al., 2006). The direct implication of CO in preserving organ function against reperfusion injury was further confirmed in a similar model of intestine preservation for transplants. Intestinal grafts stored in solutions bubbled with 5% CO gas prior to transplantation in Lewis rats displayed improved intestinal barrier function, decreased vascular resistance and reduced mucosal denudation and inflammation compared to untreated grafts (Nakao et al., 2006). These initial findings were then supported by subsequent studies demonstrating that CO delivered by CORM‐A1 protects against a wide range of pathologies including autoimmune disease (Fagone et al., 2015), diabetes (Hosick et al., 2016; Nikolic et al., 2014), liver toxicity and hepatitis (Upadhyay et al., 2018, 2020) as well as myocardial infarction (Iqbal et al., 2021).

Additional studies involving various organometallic CO carriers have further supported the potential therapeutic use of CO in treating renal dysfunction caused by ischemic events. For example, CORM‐3 has been shown to significantly alleviate the damage inflicted by ischemia–reperfusion in a porcine model of controlled non‐heart‐beating donor kidney transplantation (Bagul et al., 2008). Similarly, treatment of pig kidneys with CORM‐401 during the cold preservation phase prevented intra‐renal hemorrhage, reduced urinary protein excretion and mitigated renal damage during reperfusion ex vivo (Bhattacharjee et al., 2018). However, the therapeutic potential of CORM‐A1 as a CO pro‐drug and its ability to target ischemic kidneys has yet to be explored. We hypothesized that CORM‐A1, although capable of spontaneously releasing CO at physiological pH, could act as a CO carrier, delivering the gas in vivo and conferring protection under clinically relevant conditions. Here we demonstrate that CORM‐A1 indeed spontaneously releases CO when administered in vivo and effectively delivers this gas to the blood circulation and, most importantly, the kidney. Moreover, we present the first evidence of CORM‐A1's efficacy in alleviating post‐ischemic renal dysfunction, based on a proof‐of‐principle study conducted in both rats and swine models of kidney reperfusion injury.

## 2 MATERIALS AND METHODS

### 2.1 Chemicals and reagents

CORM‐A1 was synthesized as previously reported by our group (Motterlini et al., 2005). Stock solutions in pure distilled water (10 mM) were initially prepared and stored at −20°C until use. The cyclodextrin‐iron(II)porphyrin complex hemoCD1, which binds CO with extremely high affinity, was synthesized and used as previously described (Kitagishi et al., 2010; Mao et al., 2023). Dulbecco Phosphate Buffer Solution (DPBS, without calcium and magnesium, pH = 7.4), sodium dithionite (Na_2_S_2_O_4_) and all other reagents were purchased from Sigma Aldrich unless otherwise specified.

### 2.2 Detection of CO release from CORM‐A1 in vitro

The liberation of CO from CORM‐A1 was confirmed by measuring spectrophotometrically the formation of the hemoCD‐CO complex over time as previously reported (Mohan et al., 2023). Both the Fe(III)‐oxidized form (met‐hemoCD) and Fe(II)‐reduced form (deoxy‐hemoCD) of hemoCD1 were used for this assay. For the preparation of deoxy‐hemoCD, hemoCD1 was first solubilized in 1 mL DPBS pH = 7.4 (8 μM final concentration) using a 2 mL cuvette in the presence of 2 mg of the reducing agent Na_2_S_2_O_4_. CORM‐A1 (8 μM final concentration) was then added and spectra were immediately recorded at room temperature (24°C) between 400 and 500 nm over time to detect the appearance of the hemoCD‐CO complex (Soret peak at 422 nm) using a UV–Vis spectrophotometer (V‐730ST JASCO, France). In the case of met‐hemoCD, hemoCD1 as such was solubilized in 1 mL DPBS (10 μM) using a 2 mL cuvette in the absence of Na_2_S_2_O_4_ and CORM‐A1 (100 μM) was added before spectra were recorded over time as described above.

### 2.3 Quantification of renal CO content

Male Wistar rats (3–5 months old) were anesthetized with isoflurane (0.4 L/min) and administered intravenously (tail vein) with either saline (control group) or CORM‐A1 (20 μmoles/kg). Thirty minutes or 1 h after administration (*n* = 5 per group), animals were flushed through the femoral artery with 200 mL cold saline and kidneys collected for measuring the CO content using the hemoCD1 assay as previously described (Benrahla et al., 2024; Mohan et al., 2023). Briefly, 20 mg of renal tissue were homogenized in DPBS containing Na_2_S_2_O_4_ and hemoCD1 (2–10 μM) was added thereafter. Samples were centrifuged, supernatants filtered and collected for spectrophotometric analysis. Following the analysis of the spectra, the amount of CO was calculated based on the reported extinction coefficients for hemoCD1 at 422 and 434 nm and expressed as pmoles/mg of tissue (Benrahla et al., 2024).

### 2.4 Experimental animals

In vivo studies were performed on rats and pigs. Male Wistar rats weighing 250–300 g, sourced from Harlan Ltd., UK, were housed in groups of three per cage and fed Teklad irradiated global 16% protein rodent diet (Inotiv, USA). Large White female pigs, weighing approximately 35 kg and obtained from Davis & Davis (Haynes, Bedford, UK), were housed individually in pig pens within designated facilities, in accordance with Home Office regulations, and fed Badminton country pig nuts (Badminton Feeds, Hampshire, UK). Home Office approval was obtained through the Animals in Science Regulation Unit (ASRU), which ensures animal protection by managing compliance with the Animals (Scientific Procedures) Act 1986 (ASPA) and overseeing all ethical considerations. All experiments were conducted under licenses issued by the Home Office (UK), in accordance with the Animals (Scientific Procedures) Act 1986. Before surgery, animals were randomly divided into the groups described below with *n* = 5–6 for each group. Both rats and pigs were euthanised with an overdose of sodium pentobarbital (Nembutal, Lundbech, Denmark) administered either subcutaneously (rats) or intravenously (pigs).

### 2.5 Rat model of renal warm ischemia–reperfusion injury

The rat model of renal ischemia–reperfusion injury consisted of a temporary occlusion of the left renal artery in situ followed by contralateral nephrectomy. Specifically, rats were initially anesthetized by intramuscular injection of Hypnorm® (0.3 mL/kg) and intraperitoneal injection of diazepam (5 mg/kg). After abdominal incision, the left kidney was dissected free from the surrounding tissue and warm ischemia (WI) was induced by clamping of the left renal artery either for 45 or 60 min. At the end of the ischemic period, the clamp was removed to allow reperfusion to the left kidney while a right nephrectomy was immediately performed. The abdominal wall was then sutured, and the animals were allowed to recover. In treated animals, CORM‐A1 (5, 12.5, or 25 μmoles/kg final concentrations in saline) was administered intravenously 1 h before WI, immediately post clamp removal and at 1, 2, 3, and 4 days after reperfusion (*n* = 6 per group). Untreated animals were exposed to the same ischemic periods (45 or 60 min) and received normal saline as vehicle (*n* = 6 per group). Blood samples were collected at 1, 3, and 5 days after the ischemia–reperfusion event. Serum creatinine and urea levels were assessed by using a biochemical analyzer (Cobas Mira, Roche).

### 2.6 Pig model of renal warm ischemia–reperfusion injury

White large pigs were pre‐medicated with ketamine (5 mg/kg) and xylazine (2 mg/kg) and then anesthetized with 2% isoflurane. The abdominal area was shaved, and a skin incision was made from the xiphisternum to about 5 cm above the pubis. An incision was made into the peritoneum along the linear alba, the bowel was retracted and wrapped in a bag and aluminum foil to retain heat. This gave access to the right kidney which was dissected free of all surrounding tissue and the renal vessels exposed. The renal artery, vein and ureter were tied off and the kidney remained in situ for the chosen warm ischemic period (1 h) before the artery, vein and ureter were severed. The kidney was flushed with 50 mL Soltran solution via the renal artery and then transplanted into the left pocket where a contralateral nephrectomy had been previously performed. In treated animals (*n* = 6), CORM‐A1 (20 μmoles/kg) freshly prepared in saline was administered intravenously 1 h prior to warm ischemia and at Day 0 and after reperfusion at Days 1 and 2. Untreated animals were injected with saline. Blood samples were collected at 1, 3, 5, and 28 days after the ischemia–reperfusion event. Serum creatinine and urea levels were assessed by using a biochemical analyzer (Cobas Mira, Roche).

### 2.7 Histological analysis

All surviving animals were sacrificed on Day 28 after the ischemic insult. Kidneys were harvested, divided longitudinally, fixed in 10% formalin solution and later processed by standard methods for histological evaluation. Specimens were stained with hematoxylin and eosin (H&E) for light microscopy analysis and were graded by an experienced pathologist in a blinded manner for morphological changes. Histology images were captured at ×100 magnification unless otherwise noted in figures presented. Specimens were scored according to the following scale to assess the severity of the lesion: score 0, no lesion; score 1, minimal lesion; score 2, mild lesion; score 3, moderate lesion; score 4, severe lesion. The morphological changes analyzed in three different areas included vascular congestion, tubular and glomerular necrosis, glomerular sclerosis, inflammatory index, tubular coagulation, fibrosis and tubular cell vacuolisation.

### 2.8 Statistical analysis

Statistical analysis was carried out using two‐way analysis of variance (ANOVA) combined with the Bonferroni test. When differences were established between two groups, we used an unpaired Mann–Whitney two‐tailed *t*‐test to assess statistical significance at each time point. Data are presented as mean ± SD and differences were considered significant at *p* < 0.05.

## 3 RESULTS

### 3.1 CORM‐A1 delivers CO in vitro and in vivo

**FIGURE 1:**
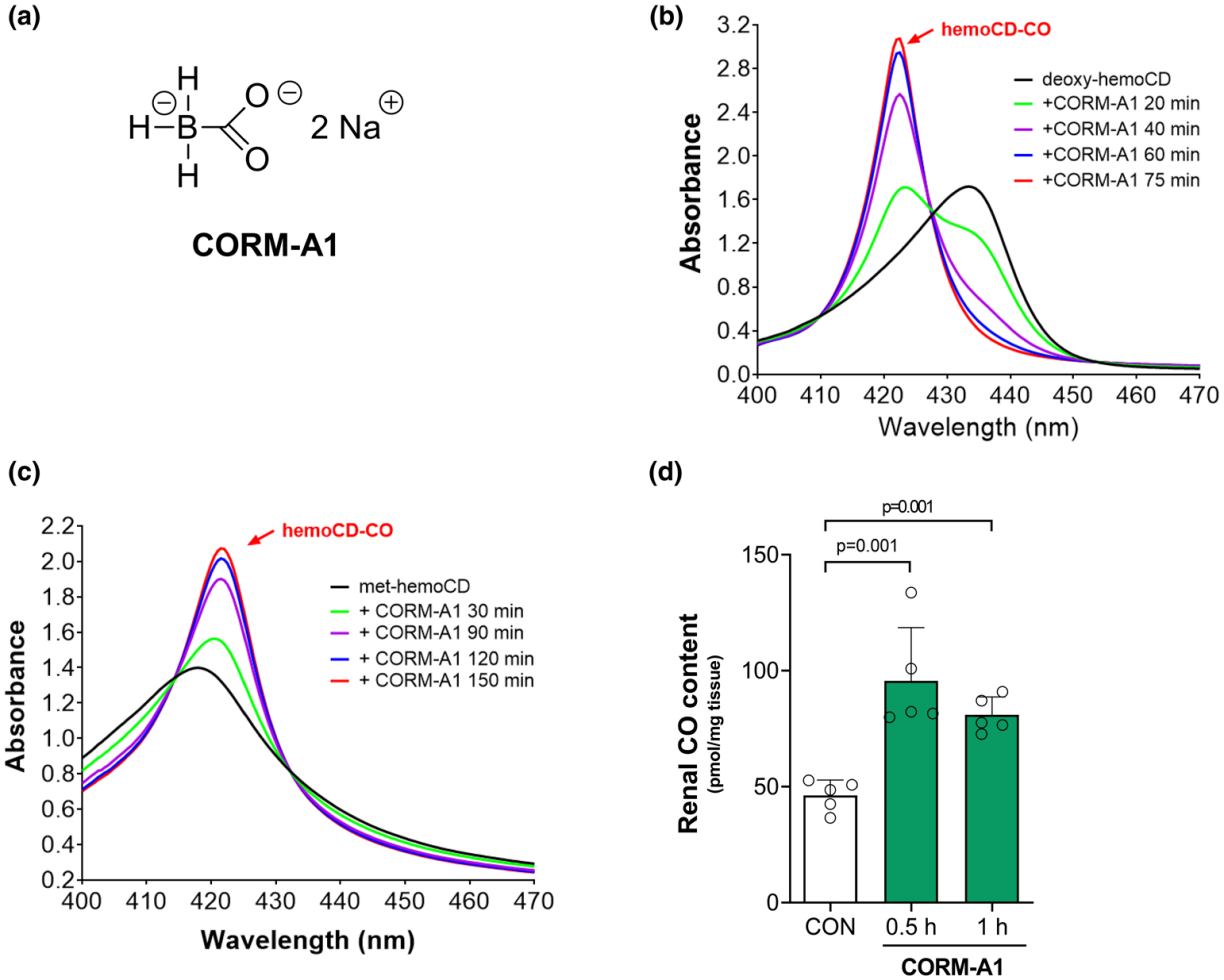
CORM‐A1 releases CO in vitro and delivers CO to the kidney after administration in vivo. The chemical structure of CORM‐A1 is represented in (a). The release of CO from CORM‐A1 in vitro was confirmed by measuring spectrophotometrically changes in the spectrum of hemoCD, a synthetic porphyrin that binds CO with extremely high affinity (see Section 2 for details). CORM‐A1 was added to a test tube containing either the reduced form deoxy‐hemoCD (b) or the oxidized form met‐hemoCD (c) and the gradual formation of a hemoCD‐CO complex (422 nm), indicative of CO release, was recorded over time. CO delivery and accumulation in renal tissue were also assessed in rats in vivo at 1 h following an intravenous infusion of CORM‐A1 (20 μmol/kg) or saline (control group). CO content in kidneys was quantified spectrophotometrically in control and CORM‐A1‐treated rats (d) after processing collected renal tissues in the presence of the CO scavenger deoxy‐hemoCD using an established protocol in our laboratory (see Materials and Methods for details). Data in the bar graph represent the mean ± SD of *n* = 5 experiments per group.

The chemical structure of CORM‐A1 is reported in Figure 1a. CO liberated from this compound at physiological pH was assessed in a test tube in vitro by measuring changes in the spectra of hemoCD1 over time between 400 and 500 nm (see Methods for details). One important piece of information to highlight about this compound is that, in addition to spontaneously delivering CO in aqueous solutions and biological media, CORM‐A1 is at the same time a reducing agent (Alberto et al., 2001). Therefore, we hypothesized that not only deoxy‐hemoCD, the reduced form of hemoCD1, but also the oxidized form met‐hemoCD would be able to bind CO providing it is first reduced by CORM‐A1 alongside its spontaneous generation of CO. This was indeed the case as confirmed by the two sets of results presented here. As shown in Figure 1b, after the addition of CORM‐A1, the characteristic absorption spectrum of deoxy‐hemoCD showing a Soret peak at 434 nm gradually shifted over time to spectra that are typical of the Fe(II)hemoCD‐CO complex, showing an increase of the absorption band at 422 nm. Similarly, the addition of CORM‐A1 to a solution containing the oxidized met‐hemoCD form (maximum absorption at 418 nm) produced over time a spectral shift toward 422 nm. These data clearly demonstrate the dual action of CORM‐A1 as an effective reducing agent and CO releaser in physiological conditions confirming previous in vitro results obtained using a myoglobin assay (Motterlini et al., 2005). To assess the ability of CORM‐A1 to release CO once administered in vivo, rats were injected intravenously with CORM‐A1 (2.06 mg/kg = 20 μmoles/kg). We found that blood carboxyhemoglobin (COHb) levels significantly increased from 0.4% at time zero to 1.7% and 8.7% after 15 and 30 min, respectively, and decreased at later times (6.9% at 120 min) after CORM‐A1 treatment (Table 1). It is interesting to note that previous studies conducted in both pigs and humans have reported that blood carboxyhemoglobin (COHb) levels of no more than 10%–12% are considered a safety threshold when using CO gas inhalation for therapeutic purposes (Hanto et al., 2010) (Fredenburgh et al., 2018). Therefore, the doses of CORM‐A1 used in our present study are justified, as they align with these findings. It is also interesting to note from the analysis of venous blood in Table 1 that the increased amount of CO in the circulation following the administration of CORM‐A1 is accompanied, as expected, by enhanced oxygen saturation (sO_2_) as well as oxygenated hemoglobin (O_2_Hb). This indicates that when animals are treated with CORM‐A1 after a period of WI, less O_2_ would be delivered to the organs during the early times of reperfusion. Finally, and most importantly, administration of CORM‐A1 resulted in a rapid (within 1 h) and marked accumulation of CO in renal tissue (Figure 1d) confirming the ability of this compound to efficiently deliver CO not only to the blood circulation but also to peripheral organs.

**TABLE 1:** Venous blood parameters following intravenous administration of CORM‐A1 (2.06 mg/kg = 20 μmol/kg) in rats.

	Time = 0 min	Time = 15 min	Time = 30 min	Time = 120 min
pH	7.18	7.18	7.26	7.27
pCO_2_ kPa	10.0	11.2	7.6	7.4
pO_2_ kPa	6.0	6.2	7.8	8.5
Hb (g/dL)	13.6	13.6	12.8	12.6
sO_2_ (%)	40.6	45.3	74.4	79.8
Hct (%)	41.9	41.8	39.3	38.7
O_2_Hb (%)	40.8	45.2	67.6	74.7
COHb (%)	0.4	1.7	8.7	6.9
HHb (%)	58.8	53.1	23.7	18.4
Total (%)	100.0	100.0	100.0	100.0

### 3.2 Effect of CORM‐A1 on renal function and kidney morphology after warm ischemia and reperfusion (WI/R) in rats

**FIGURE 2:**
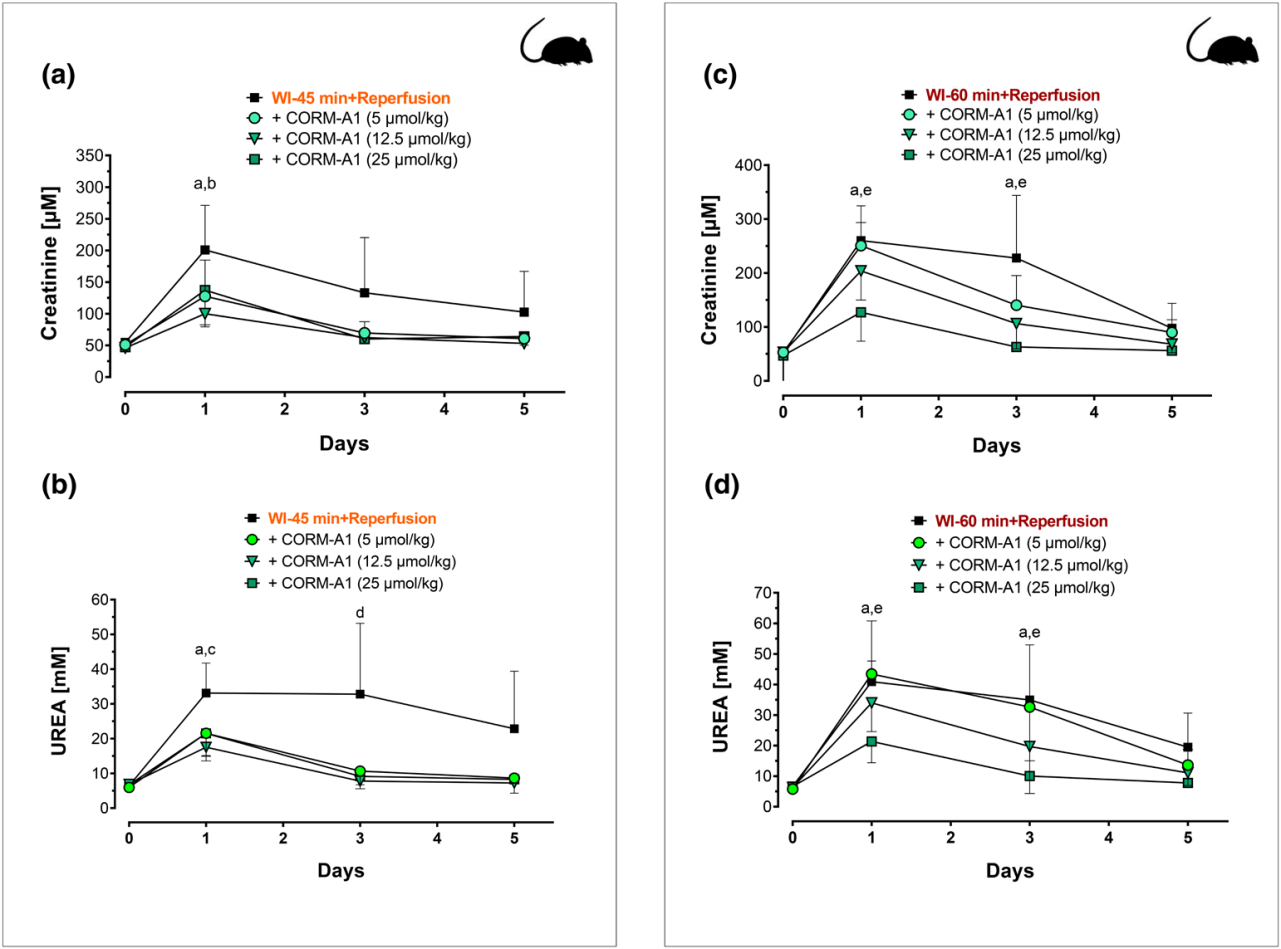
Administration of CORM‐A1 in rats protects against renal dysfunction caused by warm ischemia and reperfusion. Rats were subjected to either 45 min (panels a, b) or 60 min (panels c, d) warm ischemia (WI) followed by reperfusion as described in Materials and Methods. Serum creatinine (a and c) and urea levels (b and d) were measured at baseline and 1, 3, and 5 days after WI and reperfusion as indexes of renal function in the absence or presence of increasing concentrations of CORM‐A1. CORM‐A1 (5, 12.5, or 25 μmoles/kg) was administered intravenously 1 h before WI, immediately post clamp removal and at 1, 2, 3, and 4 days after reperfusion. Data were compared to those obtained from animals exposed to the same ischemic periods (WI‐45 min or WI‐60 min) and receiving normal saline (vehicle). Each line represents the mean ± SD of *n* = 5 for each group. a, *p* < 0.01 WI‐45 or WI‐60 min 1 day versus time 0; b, *p* < 0.01 WI‐45 min versus 12.5 μmoles /kg CORM‐A1; c, *p* < 0.03 WI‐45 min versus all CORM‐A1 groups; d, *p* < 0.01 WI‐45 min versus all CORM‐A1 groups; e, *p* < 0.01 WI‐60 min versus 25 μmoles/kg CORM‐A1.

**FIGURE 3:**
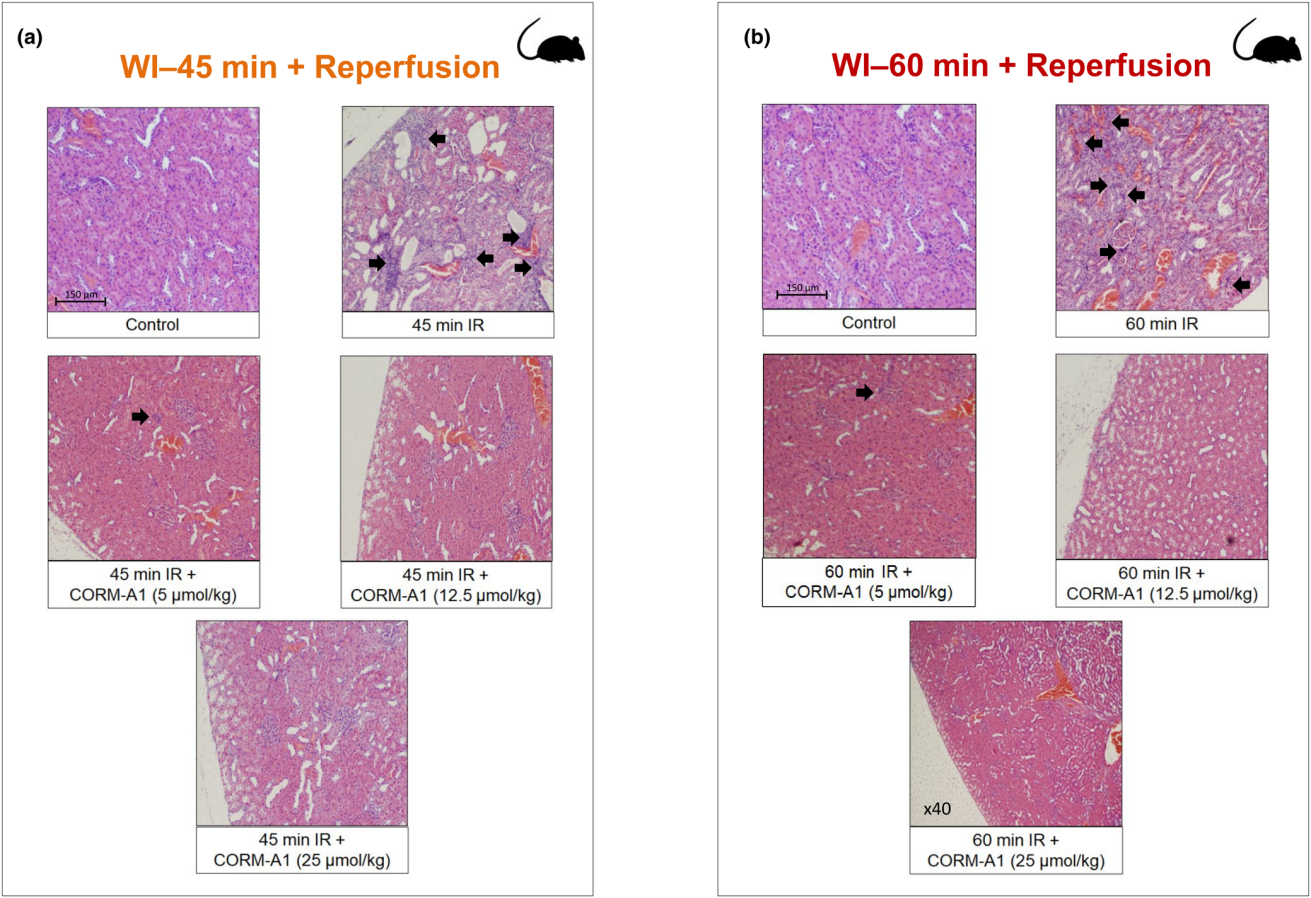
Effects of CORM‐A1 on morphological changes in rat kidneys following warm ischemia and reperfusion. Light microscopy images of kidneys collected 28 days after either WI‐45 min (a) or WI‐60 min (b) and reperfusion in the absence or presence of increasing concentrations of CORM‐A1. CORM‐A1 (5, 12.5, or 25 μmoles/kg) was administered intravenously 1 h before WI, immediately post clamp removal and at 1, 2, 3, and 4 days after reperfusion. The control group is represented by kidneys collected from rats with no ischemia. The representative micrographs reveal that kidneys subjected to both 45‐ and 60‐min ischemia–reperfusion show increased sloughing and diffuse denudation of renal tubular cells as well as acute tubular necrosis, which were significantly reduced in a dose‐dependent manner by treatment with CORM‐A1 (see Section 3 for more details). Arrows indicate infiltrated inflammatory cells. Histology images were captured at ×100 magnification, except for the 60 min IR + CORM‐A1 (25 μmol/kg) image, which was taken at ×40 magnification.

**FIGURE 4:**
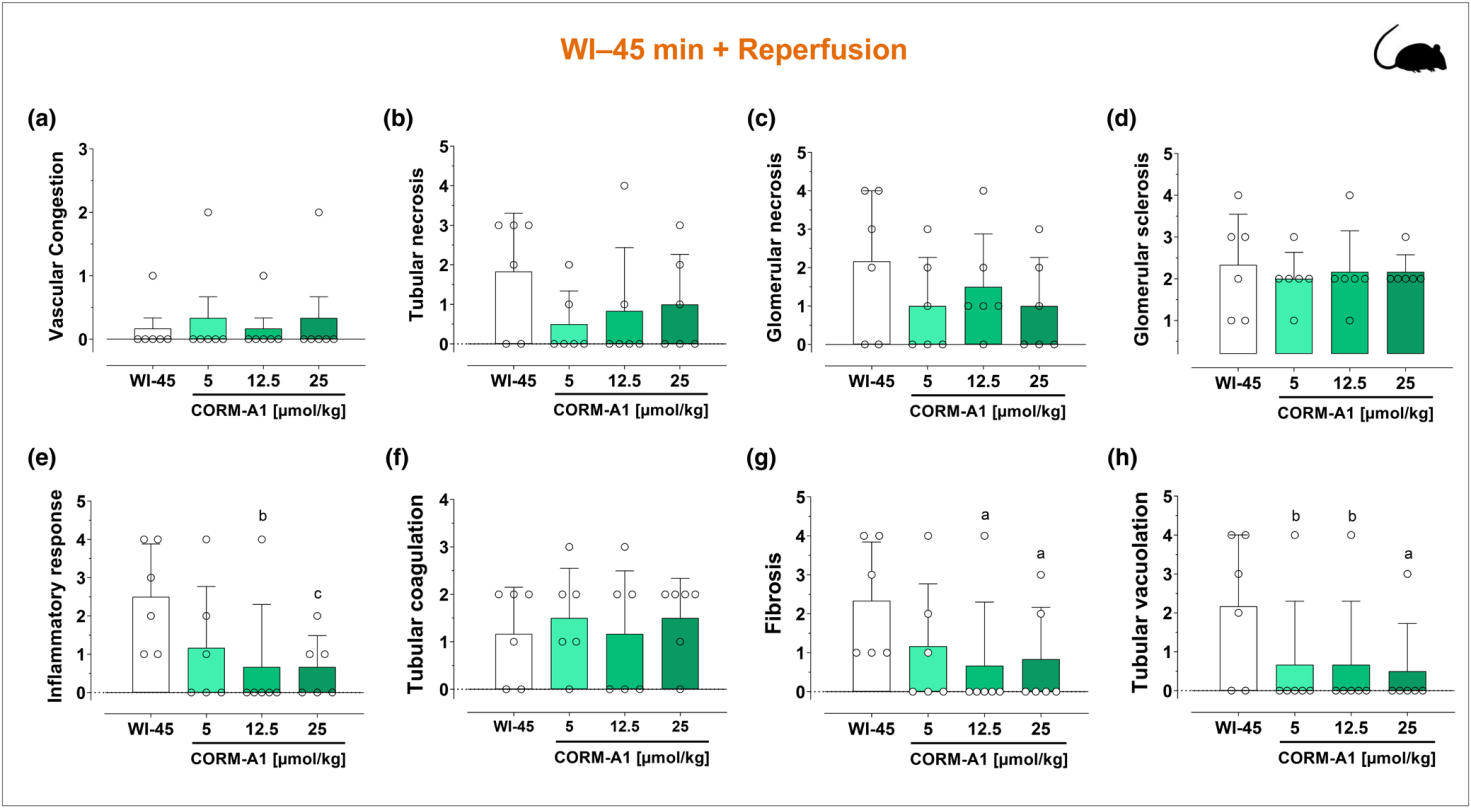
Effects of CORM‐A1 on histological parameters of rat kidneys following 45 min warm ischemia (WI) and reperfusion. Kidneys were collected 28 days after WI‐45 min and reperfusion in the absence or presence of increasing concentrations of CORM‐A1. CORM‐A1 (5, 12.5, or 25 μmoles/kg) was administered intravenously 1 h before WI, immediately post clamp removal and at 1, 2, 3, and 4 days after reperfusion. Histological specimens were scored blindly for morphological changes by light microscopy and the following parameters were evaluated: vascular congestion (a), tubular necrosis (b), glomerular necrosis (c), glomerular sclerosis (d), inflammatory response (e), tubular coagulation (f), fibrosis (g), and tubular cell vacuolisation (h) (see Sections 2 and 3 for details). Each bar represents the mean ± SD of *n* = 6 for each group. a, *p* = 0.01 WI‐45 min versus CORM‐A1; b, *p* = 0.02 WI‐45 min versus CORM‐A1; c, *p* = 0.03 WI‐45 min versus CORM‐A1.

**FIGURE 5:**
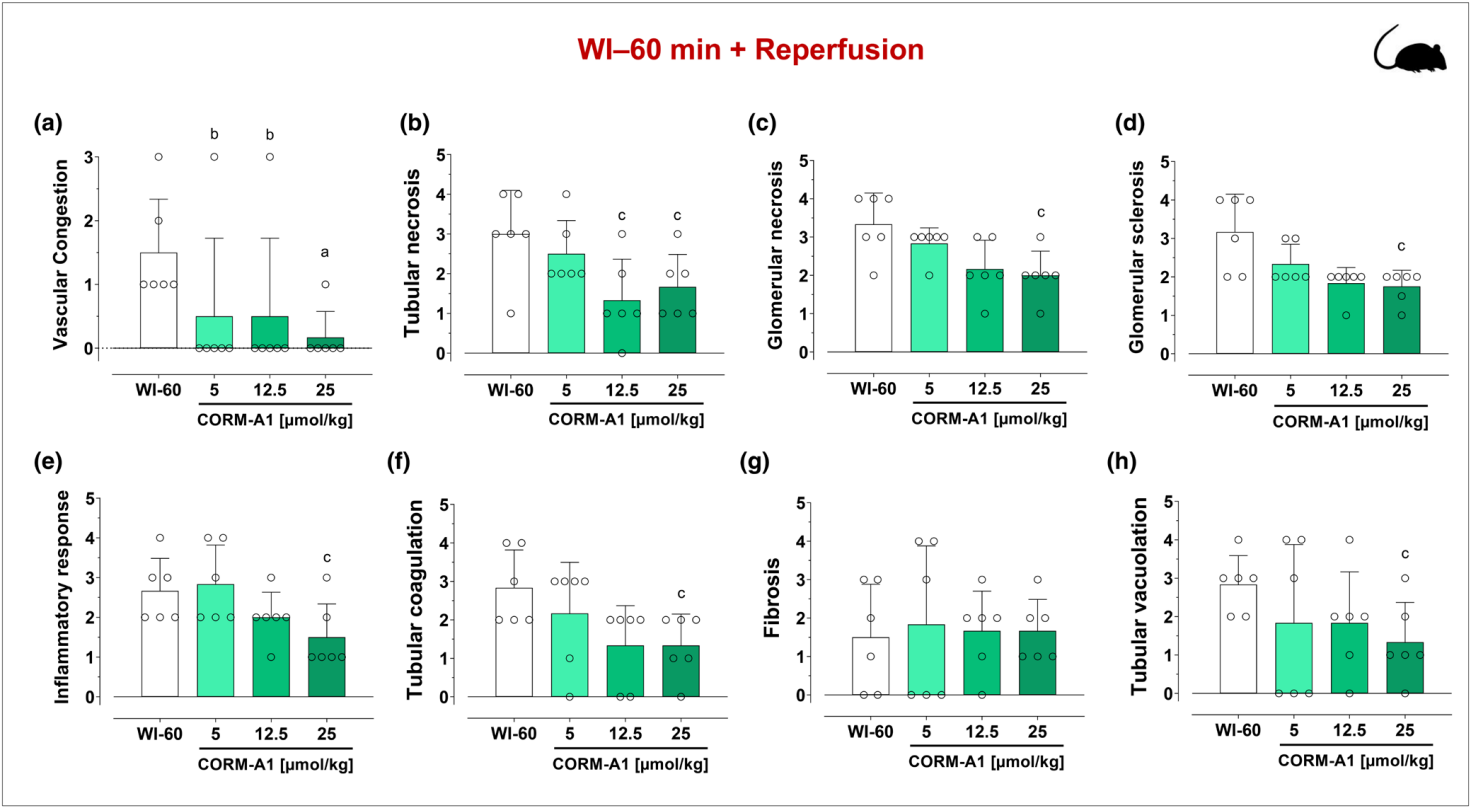
Effects of CORM‐A1 on histological parameters of rat kidneys following 60 min warm ischemia (WI) and reperfusion. Kidneys were collected 28 days after WI‐60 min and reperfusion in the absence or presence of increasing concentrations of CORM‐A1. CORM‐A1 (5, 12.5, or 25 μmoles/kg) was administered intravenously 1 h before WI, immediately post clamp removal and at 1, 2, 3, and 4 days after reperfusion. Histological specimens were scored blindly for morphological changes by light microscopy and the following parameters were evaluated: vascular congestion (a), tubular necrosis (b), glomerular necrosis (c), glomerular sclerosis (d), inflammatory response (e), tubular coagulation (f), fibrosis (g) and tubular cell vacuolisation (h) (see Sections 2 and 3 for details). Each bar represents the mean ± SD of *n* = 6 for each group. a, *p* = 0.01 WI‐60 min versus CORM‐A1; b, *p* = 0.02 WI‐60 min versus CORM‐A1; c, *p* < 0.05 WI‐60 min versus CORM‐A1.

Renal dysfunction in rats was assessed by measuring the changes in serum creatinine and urea levels at reperfusion after 45‐ or 60‐min warm ischemia (WI). As shown in Figure 2, both ischemic insults resulted in a marked increase in serum creatinine (Figure 2a,c) and urea (Figure 2b,d) levels, which were maximal at 1 day of reperfusion and gradually decreased after 3 and 5 days. Notably, these two biomarkers of renal dysfunction were significantly higher in rats subjected to 60 min WI (Figure 2c,d) compared to 45 min WI (Figure 2a,b), thus confirming the validity of these two protocols with differential degrees of acute kidney injury. Interestingly, treatment with CORM‐A1 at the onset of reperfusion resulted in a significant decrease in creatinine and urea levels after ischemia–reperfusion. Specifically, in the WI‐45 min protocol, CORM‐A1 was very effective at all doses used as the peak of creatinine and urea levels was already markedly reduced after 1 day by CORM‐A1 and both these markers of kidney damage were normalized to basal levels by Day 3 after ischemia (Figure 2a,b). In contrast, in the WI‐60 min protocol CORM‐A1 significantly decreased creatinine and urea in a dose‐dependent and time‐dependent fashion (Figure 2a,b). The histopathological analyses on a variety of renal tissue parameters confirmed that CORM‐A1 exerted protective effects against the damage inflicted by WI and reperfusion. First, the extent of kidney damage was visibly much higher after 60 min WI than 45 min WI in line with the results obtained with creatinine and urea levels. As shown in Figure 3a,b, both WI‐45 min and WI‐60 min followed by reperfusion resulted in sloughing and diffuse denudation of renal tubular cells that led to the formation of granular casts, a feature of acute tubular necrosis. This was also confirmed by widespread interstitial infiltrates and tubular atrophy after 45 min WI and to a larger extent after 60 min WI. In contrast, treatment with CORM‐A1 after 45 or 60 min WI followed by reperfusion significantly reduced in a dose‐dependent manner sloughing and denudation of renal tubular cells as well as cell infiltration. When the tissue specimens were scored blindly for specific morphological changes (see Figures 4 and 5), we found that in the WI‐45 min protocol treatment with CORM‐A1 had some degree of reduction against tubular and glomerular necrosis (Figure 4b,c) but most significantly against inflammation (Figure 4e), fibrosis (Figure 4g) and tubular vacuolation (Figure 4h). In addition, in the WI‐60 min protocol, CORM‐A1 significantly reduced vascular congestion (Figure 5a), tubular necrosis (Figure 5b), glomerular necrosis (Figure 5c) and sclerosis (Figure 5d), the inflammatory response (Figure 5e) as well as tubular coagulation (Figure 5f) and vacuolation (Figure 5h). All these effects were dose‐dependent and more effective at the highest dose used (25 μmol/kg). These results together reveal the efficacy of CORM‐A1 to protect kidneys against renal injury and dysfunction caused by WI in rats.

### 3.3 Effect of CORM‐A1 on renal function, kidney morphology, and survival in an ischemic model of kidney auto‐transplantation in swine

**FIGURE 6:**
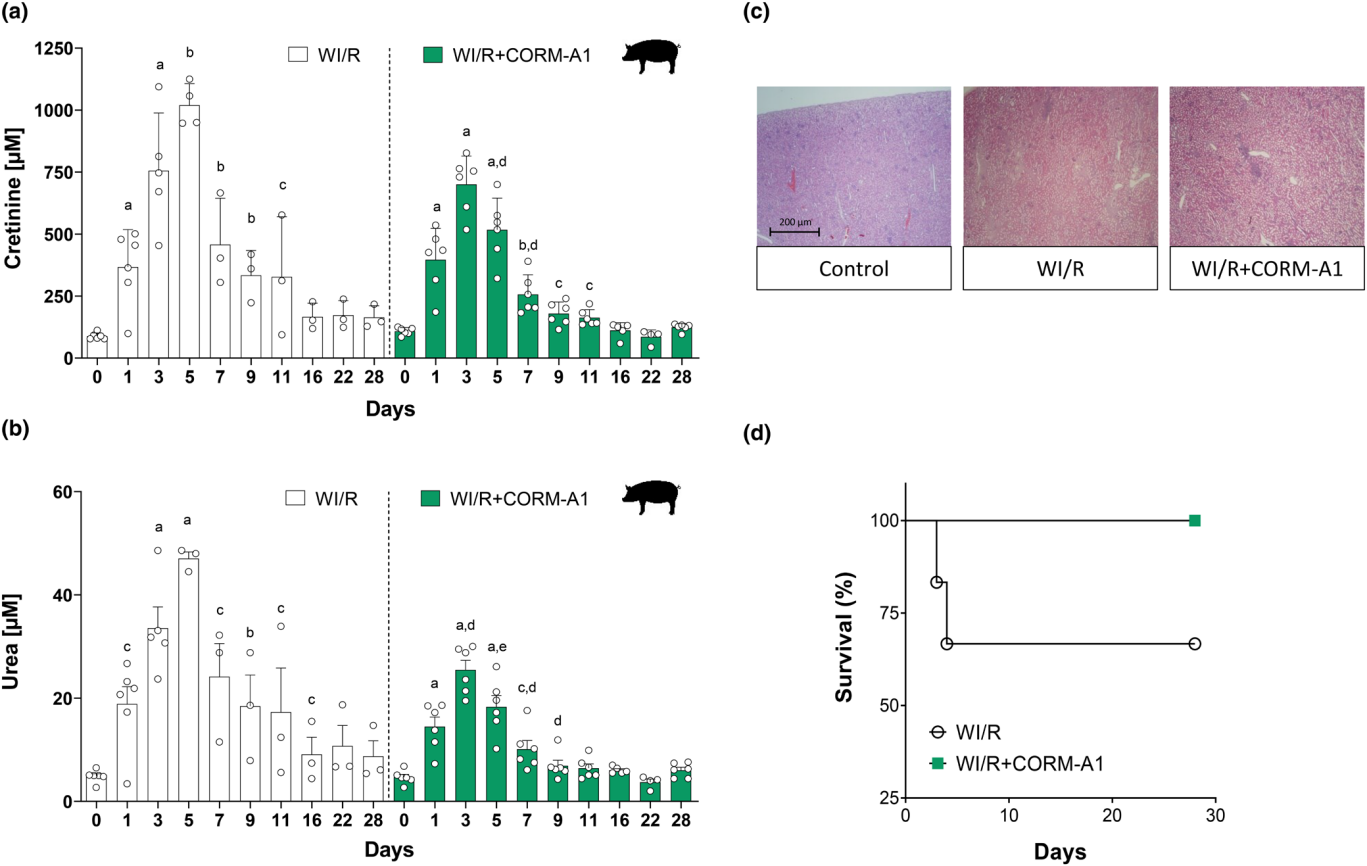
CORM‐A1 improves renal function, kidney morphology and survival in an ischemic model of kidney auto‐transplantation in swine. Pigs were subjected to 60 min warm ischemia (WI) of the right kidney, which was then removed and transplanted onto the left pocket where a contralateral nephrectomy has been previously performed (see Materials and Methods). CORM‐A1 (20 μmol/kg) was administered intravenously 1 h prior to WI and after reperfusion at Days 1 and 2. Serum creatinine (a) and urea (b) levels, indexes of renal function, were measured at baseline (time 0) and at different time points up to 28 days after reperfusion (WI/R) in the absence (white bars) or presence (green bars) of CORM‐A1 treatment. Histological analysis was performed by light microscopy on renal tissue micrographs (c) showing that CORM‐A1 preserves kidney morphology and reduces signs of fibrosis, tissue necrosis and infiltration of inflammatory cells caused by WI (see Section 3 for details). Pigs treated with CORM‐A1 also displayed an improved survival rate in this model of kidney auto‐transplantation. Each bar represents the mean ± SD of *n* = 6 for each group. a, *p* = 0.01 versus Day 0; b, *p* = 0.02 versus Day 0; c, *p* < 0.05 versus Day 0; d, *p* < 0.05 WI/R versus WI/R + CORM‐A1; e, *p* = 0.02 WI/R versus WI/R + CORM‐A1.

In the swine model, WI (60 min) of the right kidney in situ was performed similarly to the rat model but the ischemic kidney was finally removed and transplanted onto the left pocket of the same pig where a contralateral nephrectomy had been previously performed (auto‐transplantation). Inevitably, this is a more severe model of WI/R which, in addition to renal dysfunction and tissue injury, may lead to a decrease in the survival rate. We found that the increase in the levels of serum creatinine (Figure 6a, white bars) and urea (Figure 6b, white bars) were indeed significantly more pronounced compared to the ones found in rats increasing markedly at 1, 3, and 5 days of reperfusion and gradually decreasing thereafter. Notably, administration of CORM‐A1 resulted in a substantial attenuation of both creatinine (Figure 6a, green bars) and urea (Figure 6b, green bars) levels, which remained significantly reduced compared to the untreated pigs several days after reperfusion. When analyzed for their morphology 28 days after WI, it was evident that all the kidneys subjected to reperfusion displayed similar markers of damage compared to control kidneys (Figure 6c). These consisted of several areas of significant fibrosis in the cortical region of the kidney, tissue necrosis and discreet areas of inflammatory response consisting of polymorphs, macrophages and monocytes seen throughout the cortex and the medulla. These areas were often associated with a sclerotic glomerulus or a distended and slightly sclerotic tubule. In contrast, all kidneys of pigs treated with CORM‐A1 looked macroscopically normal with a slightly pale cortical surface and very minor fibrotic patches at the cortico‐medullary junction. In some kidneys there were some small to moderately sized syncitia of inflammatory cells some minor tubular distension, a few necrotic glomeruli but no evidence of tubular congestion. Overall, pig ischemic kidneys treated with CORM‐A1 showed no fibrosis, much less tissue damage and a better appearance of the glomeruli and tubular structures alongside areas with minor inflammatory cells. Finally, we found that 25% of pigs died within 6 days after WI and reperfusion, whereas the pigs receiving CORM‐A1 all survived for 28 days until they were sacrificed for analysis (Figure 6d). Altogether, these results provide the first proof‐of‐principle on the efficacy of an organic CO pro‐drug against the damage inflicted on the kidney in rodents and swine models of WI and reperfusion.

## 4 DISCUSSION

Renal ischemia is characterized by a transient or prolonged reduction in cortical blood flow causing tissue hypoperfusion and leading to functional and morphological kidney damage, which becomes more pronounced during the reperfusion phase. This condition can arise from disease states such as sepsis‐associated acute kidney injury, renal artery stenosis or during kidney transplantation (Brook et al., 2003). In the case of transplants, kidneys subjected to extended storage in preservation solutions more frequently display delayed graft function, which has been associated with a shorter lifespan of the transplanted graft (Barbosa et al., 2023; Perico et al., 2004). Although restoring blood supply is essential to revive ischemic organs, the reperfusion event itself inevitably triggers mechanisms responsible for organ dysfunction (Adams et al., 2019). In this proof‐of‐principle study we report on the efficacy of CORM‐A1, a pro‐drug that generates CO under physiological conditions, in attenuating renal dysfunction using two different animal models of warm ischemia–reperfusion (WI/R) injury. In the rat model, occlusion of the left renal artery to induce ischemia was followed by reperfusion and removal of the right kidney. Conversely, in the pig model, the ischemic right kidney was subsequently removed and transplanted into the left side, where a previous contralateral nephrectomy had been performed (auto‐transplantation). Our results showed that intravenous injections of CORM‐A1 provided significant and dose‐dependent protection against kidney injury and renal dysfunction in rats. The optimal protective dose against WI/R in this model was then used in a separate set of experiments in swine, showing improved function of transplanted kidneys and increased survival rates in animals receiving CORM‐A1. Although the translational relevance to the clinic of these findings requires further confirmation, this study demonstrates that CORM‐A1 provides strong renal protection across different animal models and types of kidney injury pathologies.

Previous pre‐clinical studies in the context of kidney preservation and transplantation have reported the beneficial actions of CO gas and CO‐RMs in attenuating tissue damage and renal dysfunction caused by ischemia–reperfusion. In one of the first studies, conducted by Neto and colleagues, renal grafts preserved in cold solutions for 24 h were transplanted into recipient rats, which were exposed to an atmosphere of CO gas (250 ppm) 1 h before and 24 h after transplantation. The results revealed that, compared to untreated control, exposure to CO gas improved serum creatinine levels and clearance, enhanced cortical blood flow, reduced macrophage infiltration and inflammatory mediators, and increased animal survival (Neto et al., 2004). Similar findings were subsequently reported using CORM‐3, a metal‐containing compound, showing that intraperitoneal administration of this compound to mice subjected to ischemia‐induced acute renal failure significantly decreased plasma creatine levels and reduced mortality (Vera et al., 2005). More recently, a single study reported that CO gas by inhalation, orally administered CO‐saturated solutions or a CO prodrug BW‐101 (given intraperitoneally at 100 mg/kg) all provided protection against acute kidney injury in mice. However, in each of these cases, mice were pre‐treated with CO before surgery and blood samples and tissues were taken for analysis only at 2, 6, and 24 h after reperfusion, thus failing to assess the true therapeutic potential of these strategies at later time points (Correa‐Costa et al., 2018). Furthermore, the study did not provide evidence that the effect of high‐dose BW‐101 was mediated by CO, as no data on blood COHb levels or CO accumulation in renal tissue were reported. In contrast, our present study demonstrates that intravenous administration of CORM‐A1 at much lower doses (25 μmoles/kg = 2.57 mg/kg) before and after reperfusion effectively reduced both creatinine and urea levels in our in vivo models of WI/R over several days following the ischemic insult, offering a more clinically robust approach for kidney preservation and transplantation.

The efficacy of CORM‐A1 in our study is validated by the robustness of the rat and pig models used, as well as the reproducibility of the data across both animal species. For example, histological analysis of kidney damage in rats showed significantly greater damage after 60 min WI compared to 45 min, consistent with the results observed in creatinine and urea levels and the expectation that a longer WI should result in exacerbated injury. In the WI‐45 min protocol, CORM‐A1 was highly effective at all doses in preventing renal dysfunction, whereas in the WI‐60 min protocol, CORM‐A1 significantly reduced creatinine and urea levels in a dose‐dependent and time‐dependent manner. These results indicate that even very low CO doses are effective in preventing smaller WI‐induced damage, while more robust doses are required when damage becomes more substantial, as in the case of WI‐60 min. This observation could be related to the extent of mitochondrial damage, since mitochondria are significantly impaired during WI/R, and we have previously shown that CORM‐3 and CORM‐A1 significantly improved mitochondrial respiration in rabbit kidneys subjected to cold preservation and reperfusion (Sandouka et al., 2006). Moreover, in both rats and pigs, treatment with CORM‐A1 alleviated several features of kidney damage, as evidenced by reduced fibrosis, less tissue damage, improved glomerular and tubular structures, and fewer inflammatory cells, suggesting multiple mechanisms of protection involved in the positive effect of CORM‐A1. Finally, in the swine model of kidney auto‐transplantation, we found that while 25% of pigs died within 6 days after WI and reperfusion, pigs receiving CORM‐A1 survived for the full 28‐day protocol. These promising results align with a previous study showing that administration of inhaled CO reduces delayed graft function in kidney allografts in swine (Hanto et al., 2010).

Our study is the first to demonstrate that CORM‐A1 treatment effectively delivers carbon monoxide (CO) in vivo, not only enhancing COHb levels in the bloodstream, as also previously described (Ryan et al., 2006), but most importantly, promoting accumulation of CO in the kidney. This delivery was rapid, occurring within 1 h of administration, and was accompanied by a notable increase in COHb levels (8.7%), which gradually decreased over time. This finding underscores the ability of CORM‐A1 to target specific organs, such as the kidney, with a controlled release of CO, highlighting its potential as a therapeutic agent for ischemic conditions. The beneficial effect of various CO‐RMs, including CORM‐A1, to significantly improve vascular function and maintain performance of organs such as the kidney, liver, and heart during cold preservation and reperfusion has been well documented (Musameh et al., 2007; Pizarro et al., 2009; Sandouka et al., 2006; Zhang et al., 2024). From these and more recent reports, it is becoming increasingly clear that CO, whether inhaled or delivered by CO‐RMs, exerts multifaceted pharmacological effects, with its therapeutic potential expanding far beyond its vasodilatory properties (Motterlini & Foresti, 2017). In the kidney, these effects include but are not limited to: (1) activation of guanylate cyclase, which enhances glomerular filtration and improves renal perfusion flow rate (Ryan et al., 2006; Sandouka et al., 2006); (2) mitigation of renal tubule apoptosis and necrosis (Hanto et al., 2010; Neto et al., 2004; Tayem et al., 2006); (3) reducing inflammation by suppressing toll‐like receptor activity, macrophage infiltration, and renal intra‐renal hemorrhage (Bhattacharjee et al., 2018; Neto et al., 2004). Collectively, these concerted mechanisms of action contribute to the preservation of kidney function during ischemia–reperfusion injury.

Over the years, our group has developed several CO‐RMs with distinct chemical structures and CO release kinetics. These are exemplified by the three most extensively studied compounds, namely: (1) the ruthenium‐based CORM‐3, which releases 1 equivalent of CO with a fast kinetic in vitro (half‐life approximately 1 min) (Clark et al., 2003); (2) the boron‐based CORM‐A1 releasing 1 equivalent of CO with a slow kinetic in vitro (half‐life approximately 20 min) (Motterlini et al., 2005); and (3) the manganese‐based CORM‐401, which releases 3 equivalents of CO in vitro with a kinetic similar to CORM‐A1 (Benrahla et al., 2024). Irrespective of their chemical reactivity and pharmacological behavior in vitro, all three compounds have shown protective actions and efficacy across different disease models. These effects have been validated using CO‐depleted CO‐RMs as negative controls to confirm the contribution of CO to the observed effects. One potential advantage of CORM‐A1 over other CO‐RMs is its immediate pharmacological effects following intravenous administration, due to its ability to spontaneously generate CO from its COOH group at physiological pH within the blood circulation and its diffusion to tissues.

In summary, intravenously administered CORM‐A1 demonstrates consistent efficacy in reducing kidney injury across multiple WI/R models, underscoring the potential of controlled CO delivery as a kidney‐protective therapy to improve renal function in pathological conditions. While several alternative organic CO prodrugs have been developed in recent years, so far none have undergone comprehensive characterization or rigorous preclinical evaluation, and conclusive evidence supporting their CO‐mediated therapeutic effects is lacking (Ji & Wang, 2018). Some recent unfounded and misleading critiques have been raised by a group of chemists who questioned the extent and consistency of CO release from CORM‐A1 and other related metal‐based CO‐RMs developed by our group, as well as their CO‐mediated pharmacological action. Rather than reiterating unsubstantiated claims on the CO‐RMs used by us, these groups might more productively direct their efforts toward experimentally validating the efficacy of the organic CO prodrugs they have so far reported only through sporadic and fragmentary data. In fact, their questionable claims on the CO‐RMs we have developed were based solely on oversimplified in vitro experiments they conducted by reacting these compounds with selected metabolites in physiological buffer solutions in test tubes (not even in cellular models) (Bauer, Yang, et al., 2023; Bauer, Yuan, et al., 2023), which fail entirely to capture the complex biochemical context in which these compounds operate as potential therapeutics in vivo. It is highly surprising that this group of chemists continues to maintain the untenable assumption that drugs can be engineered to bind selectively to a single target without eliciting any off‐target effects, overlooking that such effects might provide additional therapeutic benefits. Ultimately, the diversified chemical reactivity of CO‐RMs as well as the liberation of CO in complex biological systems, such as living organisms affected by diseases, constitutes the only experimental model that truly matters in drug testing and therapeutic development. Importantly, our extended in vivo studies, supported by independent research from others, clearly demonstrate that CORM‐A1 and other CO‐RMs, but not their inactive counterparts, efficiently deliver CO and exert significant therapeutic effects against different disorders (Bagul et al., 2008; Benrahla et al., 2024; Bhattacharjee et al., 2018; Clark et al., 2003; Fagone et al., 2015; Iqbal et al., 2021; Lancel et al., 2009; Motterlini et al., 2005; Motterlini & Foresti, 2017; Nguyen et al., 2024; Tayem et al., 2006; Upadhyay et al., 2018, 2020). The present findings reinforce the promise of CORM‐A1 as the most viable and efficacious among currently available CO pro‐drugs, highlighting the need for continued investigation on CO‐based therapies within physiologically relevant systems to better understand and optimize their clinical potential.

## AUTHOR CONTRIBUTIONS

Roberta Foresti and Roberto Motterlini conceived and designed research. Roberta Foresti, Colin J. Green, and Roberto Motterlini secured funding for the study. Sandra Shurey, Qiyue Mao, Hiroaki Kitagishi, Roberta Foresti, and Roberto Motterlini performed experiments. Sandra Shurey, Roberta Foresti, and Roberto Motterlini analyzed the data and prepared figures. Roberta Foresti and Roberto Motterlini drafted the manuscript. Roberta Foresti, Colin J. Green, Sandra Shurey, Qiyue Mao, Hiroaki Kitagishi, and Roberto Motterlini edited, revised and approved the final version of the manuscript.

## FUNDING INFORMATION

This work was supported by funding from *hemoCORM Ltd*. and the Kidney Research UK (Roberto Motterlini and Roberta Foresti).

## CONFLICT OF INTEREST STATEMENT

Roberto Motterlini is a former Founder and Member of the Board of Directors of *hemoCORM Ltd*. Roberto Motterlini and Roberta Foresti have filed patents for the use of CO‐RMs as therapeutics against gastrointestinal, metabolic, and inflammatory disorders.

## Data Availability

The data that support the findings of this study are available from the corresponding author upon reasonable request.
